# Non-rapid eye movement sleep slow-wave activity features are associated with amyloid accumulation in older adults with obstructive sleep apnoea

**DOI:** 10.1093/braincomms/fcae354

**Published:** 2024-10-07

**Authors:** Diego Z Carvalho, Vaclav Kremen, Filip Mivalt, Erik K St. Louis, Stuart J McCarter, Jan Bukartyk, Scott A Przybelski, Michael G Kamykowski, Anthony J Spychalla, Mary M Machulda, Bradley F Boeve, Ronald C Petersen, Clifford R Jack, Val J Lowe, Jonathan Graff-Radford, Gregory A Worrell, Virend K Somers, Andrew W Varga, Prashanthi Vemuri

**Affiliations:** Division of Pulmonary and Critical Care Medicine, Department of Internal Medicine, Center for Sleep Medicine, Rochester, MN 55905, USA; Department of Neurology, Mayo Clinic, Rochester, MN 55905, USA; Department of Neurology, Mayo Clinic, Rochester, MN 55905, USA; Department of Neurology, Mayo Clinic, Rochester, MN 55905, USA; Division of Pulmonary and Critical Care Medicine, Department of Internal Medicine, Center for Sleep Medicine, Rochester, MN 55905, USA; Department of Neurology, Mayo Clinic, Rochester, MN 55905, USA; Division of Pulmonary and Critical Care Medicine, Department of Internal Medicine, Center for Sleep Medicine, Rochester, MN 55905, USA; Department of Neurology, Mayo Clinic, Rochester, MN 55905, USA; Department of Cardiovascular Medicine, Mayo Clinic, Rochester, MN 55905, USA; Department of Quantitative Health Sciences, Mayo Clinic, Rochester, MN 55905, USA; Department of Information Technology, Mayo Clinic, Rochester, MN 55905, USA; Department of Radiology, Mayo Clinic, Rochester, MN 55905, USA; Department of Psychiatry and Psychology, Mayo Clinic, Rochester, MN 55905, USA; Division of Pulmonary and Critical Care Medicine, Department of Internal Medicine, Center for Sleep Medicine, Rochester, MN 55905, USA; Department of Neurology, Mayo Clinic, Rochester, MN 55905, USA; Department of Neurology, Mayo Clinic, Rochester, MN 55905, USA; Department of Radiology, Mayo Clinic, Rochester, MN 55905, USA; Department of Radiology, Mayo Clinic, Rochester, MN 55905, USA; Department of Neurology, Mayo Clinic, Rochester, MN 55905, USA; Department of Neurology, Mayo Clinic, Rochester, MN 55905, USA; Department of Cardiovascular Medicine, Mayo Clinic, Rochester, MN 55905, USA; Division of Pulmonary, Critical Care and Sleep Medicine, Icahn School of Medicine at Mount Sinai, New York, NY 10029, USA; Department of Radiology, Mayo Clinic, Rochester, MN 55905, USA

**Keywords:** sleep, slow-wave activity, obstructive sleep apnoea, amyloid, Alzheimer’s disease

## Abstract

Obstructive sleep apnoea (OSA) is associated with an increased risk for cognitive impairment and dementia, which likely involves Alzheimer’s disease pathology. Non-rapid eye movement slow-wave activity (SWA) has been implicated in amyloid clearance, but it has not been studied in the context of longitudinal amyloid accumulation in OSA. This longitudinal retrospective study aims to investigate the relationship between polysomnographic and electrophysiological SWA features and amyloid accumulation. From the Mayo Clinic Study of Aging cohort, we identified 71 participants ≥60 years old with OSA (mean baseline age = 72.9 ± 7.5 years, 60.6% male, 93% cognitively unimpaired) who had at least 2 consecutive Amyloid Pittsburgh Compound B (PiB)-PET scans and a polysomnographic study within 5 years of the baseline scan and before the second scan. Annualized PiB-PET accumulation [global ΔPiB(log)/year] was estimated by the difference between the second and first log-transformed global PiB-PET uptake estimations divided by the interval between scans (years). Sixty-four participants were included in SWA analysis. SWA was characterized by the mean relative spectral power density (%) in slow oscillation (SO: 0.5–0.9 Hz) and delta (1–3.9 Hz) frequency bands and by their downslopes (SO-slope and delta-slope, respectively) during the diagnostic portion of polysomnography. We fit linear regression models to test for associations among global ΔPiB(log)/year, SWA features (mean SO% and delta% or mean SO-slope and delta-slope), and OSA severity markers, after adjusting for age at baseline PiB-PET, *APOE ɛ4* and baseline amyloid positivity. For 1 SD increase in SO% and SO-slope, global ΔPiB(log)/year increased by 0.0033 (95% CI: 0.0001; 0.0064, *P* = 0.042) and 0.0069 (95% CI: 0.0009; 0.0129, *P* = 0.026), which were comparable to 32% and 59% of the effect size associated with baseline amyloid positivity, respectively. Delta-slope was associated with a reduction in global ΔPiB(log)/year by −0.0082 (95% CI: −0.0143; −0.0021, *P* = 0.009). Sleep apnoea severity was not associated with amyloid accumulation. Regional associations were stronger in the pre-frontal region. Both slow-wave slopes had more significant and widespread regional associations. Annualized PiB-PET accumulation was positively associated with SO and SO-slope, which may reflect altered sleep homeostasis due to increased homeostatic pressure in the setting of unmet sleep needs, increased synaptic strength, and/or hyper-excitability in OSA. Delta-slope was inversely associated with PiB-PET accumulation, suggesting it may represent residual physiological activity. Further investigation of SWA dynamics in the presence of sleep disorders before and after treatment is necessary for understanding the relationship between amyloid accumulation and SWA physiology.

## Teaser

Carvalho *et al.* studied slow-wave activity features in non-rapid eye movement sleep of older adults with obstructive sleep apnoea. Contrary to their hypothesis, they found that an increase in slow oscillations (0.5–0.9 Hz) and their slow-wave slopes were associated with increased longitudinal amyloid PET accumulation.

## Graphical Abstract

**Graphical Abstract:**
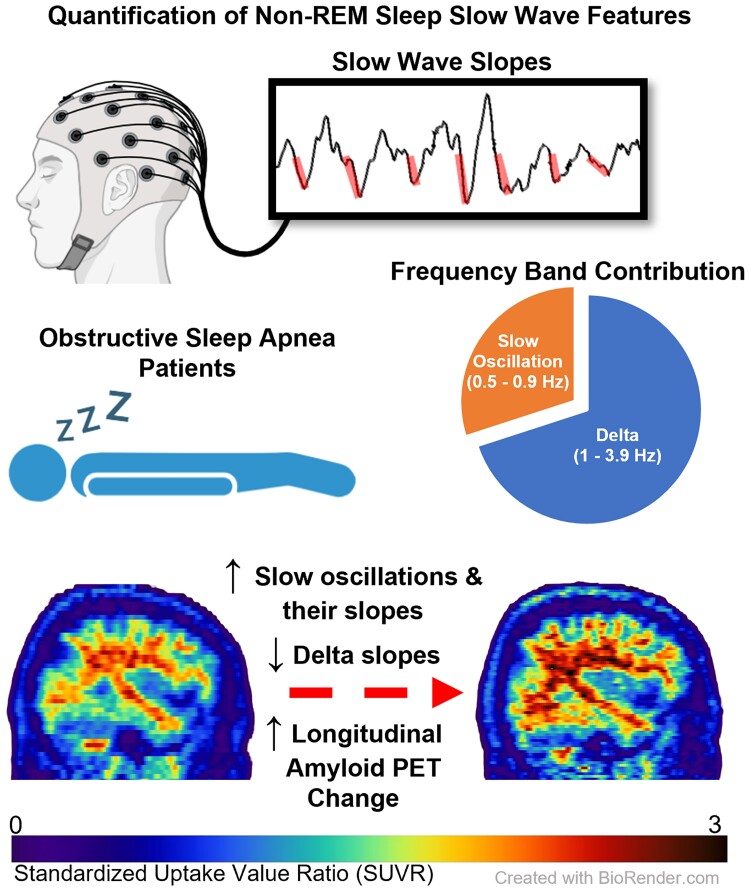

## Introduction

Obstructive sleep apnoea (OSA) is characterized by recurrent episodes of shallow breathing or breathing pauses during sleep in the setting of transient airway narrowing or collapse, respectively. The pooled prevalence of OSA from 39 studies (*n* = 33 353) was estimate at 35.9% (95% CI: 28.7–43.8%) in older adults (age ≥ 65 years old),^1^ reaching up to 70% in nursing home residents.^2^ OSA is associated with an increased risk for cognitive impairment and dementia,^3-6^ but mechanisms underlying these associations have not been elucidated.^6-10^

OSA-related cognitive decline may involve Alzheimer’s disease pathology,^6,8,10^ namely β-amyloid (Aβ) and tau, which start to accumulate years before cognitive changes.^11^ Despite progress in identifying associations between OSA (diagnosis,^12-17^ severity parameters^18-21^ or symptoms^22,23^) and Alzheimer’s disease biomarkers, longitudinal studies are scarce.^15,19^ Although it has been hypothesized that the relationship between OSA and Alzheimer’s disease may be in part related to reduction/disruption of non-rapid eye movement (NREM) Stage 3 [N3 or slow-wave sleep (SWS)] and/or NREM sleep slow-wave activity (SWA),^8,24^ it remains unclear how these features are associated with Alzheimer’s disease biomarkers in the context of OSA.^13,25^

SWA represents synchronized cortical network fluctuations of down (hyper-polarized or quiescent) and up (depolarized or active) states.^26,27^ SWA is generally characterized by the contribution of frontally predominant delta activity in the 0.5–4 Hz frequency range during NREM sleep,^13,28-30^ but frequency-specific mechanisms may exist.^26^ While some studies have found associations between SWA with cognitive function^31,32^ and Aβ^33,34^ within slower frequency ranges (<1 Hz) known as slow oscillations (SOs),^26,27,35^ another study found an association between Alzheimer’s disease biomarkers and SWS within higher frequency ranges (1–4 Hz),^25^ known as delta (or delta waves).^26^ In addition to its frequency properties, SWA can also be characterized by morphological features, including slopes of slow waves (SWs).^36^ The slopes represent the rate of neuronal decruitment (downslope) and recruitment (upslope), following or preceding SW up states, respectively.^37,38^ SW slopes have been implicated in homeostatic sleep regulation along with SWA,^36,37,39^ but it remains unknown whether a possible relationship between SW slopes and Alzheimer’s disease pathophysiology exists.

In this study, we investigated the relationship between baseline diagnostic polysomnographic (PSG) and electrophysiological SWA features (different frequency ranges and SW slopes) with longitudinal Pittsburgh Compound B (PiB) PET (amyloid) accumulation in older adults with OSA. We hypothesized that (i) higher OSA severity, (ii) decreased N3 sleep and/or SWA in different frequency ranges and (iii) decreased SW slopes would be associated with greater global and regional Aβ accumulation.

## Materials and methods

### Participants

This longitudinal retrospective study enrolled participants of the Mayo Clinic Study of Aging (MCSA) cohort, who were also seen at our Mayo Clinic Center for Sleep Medicine for clinical care. The MCSA is a large population-based cohort of community-dwelling residents from Olmsted County (Minnesota), which began in 2004 and was derived from the Rochester Epidemiology Project. Further details of MCSA design are available elsewhere.^40^ Out of all 9472 patients aged 60 and above who underwent PSG at Mayo Clinic between 2010 and 2018, 8834 patients did not have a diagnosis of dementia or other neurodegenerative disorder at the time of the sleep study. Of these, 7841 were found to have OSA. We then identified 86 patients who (i) were participants of the MCSA, (ii) completed at least 2 PiB-PET scans as part of the MCSA protocol and (iii) did not have dementia at baseline scan. We included 71 of these patients who had a PSG within 5 years of baseline scan and before the second scan. For SWA analyses, we excluded seven participants due to unavailable/poor-quality raw EEG data. This study was approved by the institutional review boards of the Mayo Clinic and Olmsted Medical Center, and written informed consent was obtained from all participants.

### Cognitive assessment

The clinical diagnoses (cognitively unimpaired [CU], mild cognitive impairment [MCI] or dementia) of the participants at baseline and follow-up were determined by consensus agreement among the MCSA study coordinator, neuropsychologist and examining physician according to published criteria, as previously described.^40,41^

### Clinical assessment

The history of medical comorbidities was abstracted by trained nurses from the Rochester Epidemiology Project medical records linkage system.^42^ We also obtained *apolipoprotein E* (*APOE*) genotyping, as previously described.^43^ Participants with one or more *APOE ɛ4* alleles were considered to have a positive (+) *APOE ɛ4* status.

### Imaging assessment

Amyloid burden was measured by PiB-PET scan with 11(C-PiB).^44^ It consisted of four 5-min dynamic frames performed 40–60 min after injection. PiB-PET data were analysed at each time point with our in-house fully automated image processing pipeline, as previously described.^23,45,46^ In brief, image processing included (ii) co-registering the PiB to structural MRI and (ii) sharpening (exclusion of voxels with a higher probability of being CSF compared with the probability of grey and white matter combined). Regional PiB-PET uptake was obtained from bilateral Aβ-susceptible regions of interest (ROIs),^46,47^ which included the orbitofrontal, pre-frontal, anterior cingulate (anterior and mid-cingulate), posterior cingulate/precuneus, parietal and temporal regions. We computed the regional PiB-PET standardized uptake value ratio (SUVR) by estimating the median uptake in each of the ROIs and normalizing them by the median uptake in the cerebellar crus grey matter.^48^ For global PiB estimation at each time point, we calculated the weighted average PiB SUVR from the ROIs under study. Global PiB positivity (PiB+) was defined by an SUVR ≥1.48 at the baseline scan. This cut-off is equivalent to our previously defined cut-off of 1.42 based on the reliable worsening method,^49^ after adjustment for updated processing pipelines and reference regions (cerebellar hemisphere to cerebellar crus). This is a reliable cut-off to select participants in whom Aβ accumulation is more likely to occur^50^ and corresponds to Thal amyloid phases of at least 1–2.^51^

Our primary outcome measure was annualized global PiB accumulation, hereafter referred to as global ΔPiB SUVR(log)/year. It was estimated by calculating the difference between the second and first log-transformed global PiB uptake SUVRs, divided by the interval between the two PiB-PET scans in years. Annualized regional PiB accumulation [e.g. frontal ΔPiB SUVR(log)/year] was estimated in similar fashion for each ROI. For direct comparison with other studies using amyloid PET, we included models with global ΔPiB measured by a difference between centiloid unit (CL)–converted SUVRs, based on previous work.^52^ The relationship between our PiB SUVR values and PiB CL value is described by the following equation: PiB.CL = 100 × [(−0.1620 + 0.9467 × PiB.SUVR)−1.009)/1.067]. Regional associations are reported only for SUVR because standardization to CL values has not been defined for regional amyloid PET data.

### Sleep assessment

All patients were referred to our Center for Sleep Medicine at Mayo Clinic for sleep complaints and were interviewed by a sleep medicine provider between 2010 and 2018, as part of clinical care. Sleep apnoea treatment with positive airway pressure (PAP) information was manually abstracted from medical records. Effective PAP therapy was defined based on documentation from the sleep visit closest to the second PiB-PET scan. PAP therapy was considered effective if there was a usage of PAP for at least 70% of nights for more than 4 h and with apnoea–hypopnea index (AHI) ≤5 per hour in the absence of excessive leaks as recorded by the PAP machine or based on the clinical judgement of the sleep provider who lastly assessed the patient when PAP data were not available. Only one patient was on treatment with a PAP alternative (oral appliance therapy), for which the effectiveness of the therapy could not be determined.

The PSG data were obtained from split-night studies performed as part of clinical care. Sleep stages and breathing events were scored visually following the American Academy of Sleep Medicine scoring guidelines.^53^ Breathing event scoring followed the initially recommended (Rule 4A),^54^ then acceptable (Rule 1B),^53,55^ hypopnoea scoring rule, which accounts for events with a reduction in flow amplitude of at least 30% for a minimum of 10 s, terminating in a ≥4% drop in oxygen saturation or arousal. Our split-night protocol consisted of a diagnostic portion followed by a PAP titration portion. During the diagnostic portion, sleep was observed without intervention. Real-time visual scoring of breathing events was performed for at least 120 min. Termination of this portion required the presence of an AHI of at least five events per hour, unless the study was designed otherwise as per provider discretion. For this study, analyses were focused on PSG parameters obtained during the diagnostic portion, consistent with the representation of each participant’s sleep architecture and apnoea severity before OSA treatment. Sleep apnoea severity was classified based on the AHI as mild (5/h ≥ AHI < 15/h), moderate (15/h ≥ AHI < 30/h) or severe (AHI ≥ 30/h).

PSG recordings were conducted on a 16-channel Nicolet NicVue digital system with sensitivity at 5–7 μV/mm. EEG was initially bandpass filtered from 0.3 to 100 Hz (Cardinal Health Corporation, Madison, WI, USA) and digitized at a sampling rate of 500 Hz. EEG recordings were performed according to the International 10–20 system electrode placements (Fp1, Fp2, Fpz, Fz, Cz, C3, C4, O1, O2 and Oz), including electrooculography, sub-mentalis and anterior tibialis EMG and an electrocardiogram. Respirations were analysed using an oronasal thermistor and nasal pressure sensor for airflow monitoring, with thoraco-abdominal impedance plethysmography to monitor effort. Oxyhaemoglobin saturation (SpO_2_) was evaluated by pulse oximetry.

### Slow-wave features extraction

**Figure 1:**
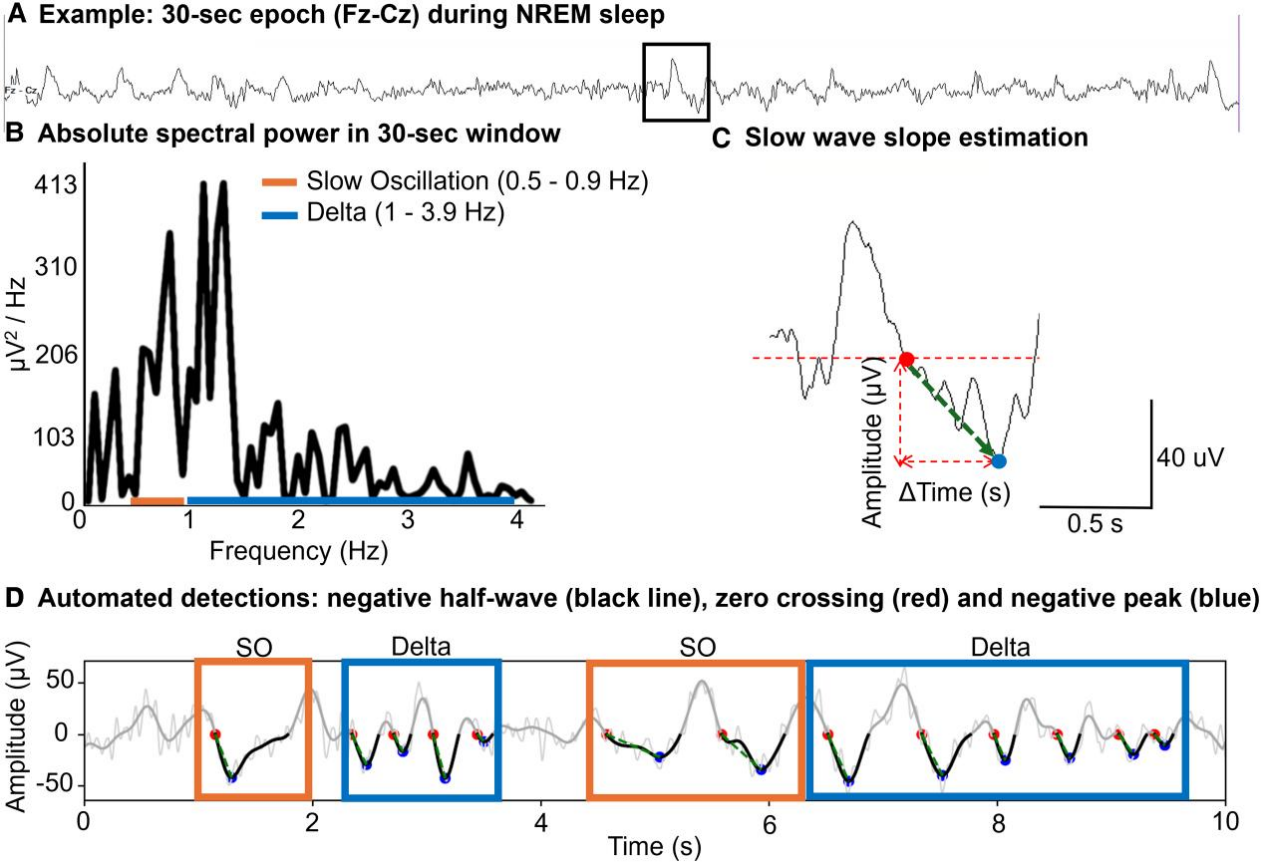
**Slow-wave feature extraction.** For every 30-s window of visually scored NREM sleep (N3 in the example) **(A)** during diagnostic portion of PSG, PSD in each frequency band of interest (e.g. SO and delta) was obtained **(B)** along with their SW slopes **(C)**. **(D)** Illustrates automated detections of negative half-waves (black lines), zero-crossings (red dot), negative peaks (blue dots) and descending slopes (diagonal green dashed line from zero-crossing to negative peak) in both SO and delta waveforms.

**Figure 2:**
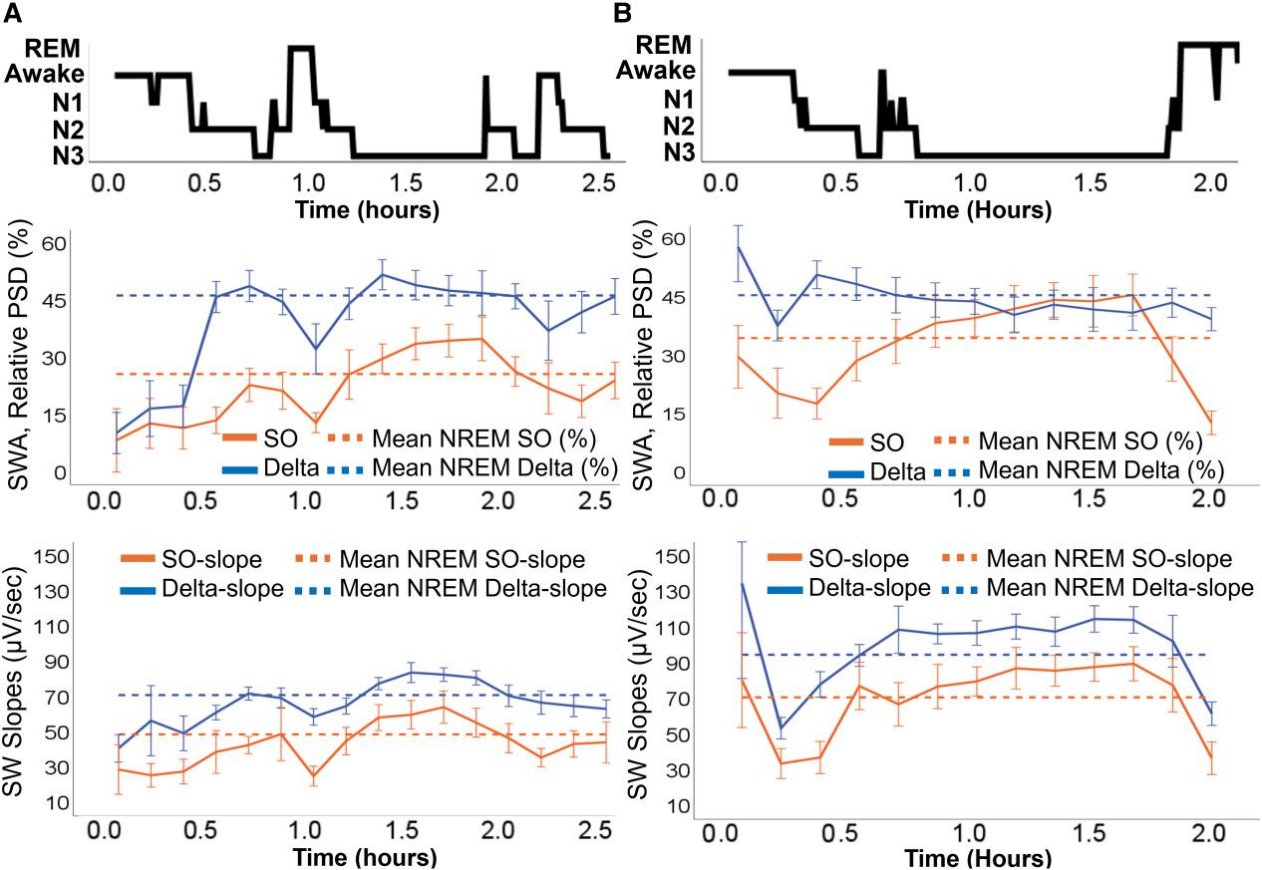
**Slow-wave feature summarization. (A)** and **(B)** Illustrate two hypnograms (top) from patients with similar sleep architecture despite different rates of PiB accumulation (**A**—24% tile of accumulation: 72 years old, *APOE ɛ4*[−], PiB[−], AHI 16/h and **B**—97% tile of accumulation: 64 years old, *APOE ɛ4*[ + ], PiB[ + ], AHI 8/h), followed by their different contributions of SWA (%) as measured by the relative PSD in SO (0.5–0.9 Hz) and delta (1–3.9 Hz) frequency bands (middle) and by their respective slow-wave slopes (bottom). Continuous lines represent relative spectral power and slopes, respectively, in each frequency band (orange = SO; dark blue = delta) averaged out for every 10 min of sleep with 95% CI of their mean (for easier visualization of trends relative to sleep stages). Dashed lines represent their means across all NREM sleep epochs.

The PSG EEG data were exported as European Data Format files and analysed using custom algorithms developed in Python programming language (Python 3.8) to characterize EEG features. Spectral power in Fz-Cz derivation was obtained for specific frequency bands of interest in each 30-s epochs (Fig. 1A), following a previously described approach using Fast Fourier Transform^56^ and consistent with previous literature.^13,29,33^ We first applied a bandpass finite impulse response filter with zero phase shift to the bandpass signal in the 0.5–35 Hz band to extract spectral power features. The filter length was 2000, and we used a hamming window design method. A power spectral density (PSD) related to SWA was divided into two frequency bands, similar to other studies:^26,33^ SO (0.5–0.9 Hz) and delta (1–3.9 Hz; Fig. 1B). We also obtained the spectral power in theta (4–7.9 Hz), alpha (8–11.9 Hz), sigma (12–14.9 Hz), beta (15–29.9 Hz) and low gamma (30–35 Hz) activity to estimate a relative PSD (% contribution), i.e. the division of the power within a frequency band of interest (e.g. SO) by the sum of the power in all frequency bands under study. After obtaining the relative PSD (%) for SO and delta bands for each 30-s epoch, we summarized the data by calculating the mean SO% and mean delta% across all visually scored NREM sleep (N1–N3) epochs (Fig. 2A and B, middle panels).

We also obtained SW slopes as defined by the downslope of the negative half-wave (Fig. 1C), similar to other studies.^36,39^ For artefact-free NREM sleep data segments, we first filtered the signal by a bandpass finite impulse response filter with zero phase shift into 0.5–35-Hz band (same as above). Subsequently, we applied a 50-ms moving average filter.^39^ Then, we automatically detected zero-crossings on the signal and selected the descending segments of negative half-waves whose zero-crossings were separated by 1.1–2 s for SO and 0.25–1.0 s for delta waves (Fig. 1D). Subsequently, we automatically identified negative peaks (troughs) of SWs after each zero-crossing on the signal. As a final step, we divided the amplitude difference (zero-crossing to negative peak in µV) by their interval (time difference between them in seconds; Fig. 1C and D). Although seminal work on slope estimations did not include a SW amplitude threshold,^39^ we followed an approach similar to other studies with a SW amplitude threshold set at −5 µV^57^ to eliminate waveforms we were not confident represented SWA. This amplitude threshold was shown to be more sensitive to detect homeostatic pressure changes than higher cut-offs.^57^ Higher amplitude thresholds can significantly bias SW detections and slope measurements in older adults, with smaller SW amplitudes.^58^ For better SW resolution (due to larger inter-electrode distance) and to generate slope estimates comparable with previous literature using ear lobe reference,^36,39,57^ we detected SWs and measured slopes in Fz referenced to ear lobe average, i.e. (A1 + A2)/2. Summary slope metrics were obtained by averaging the SW slopes in SO and delta bands in each 30-s epoch and then across all NREM epochs, generating a mean SO-slope and mean delta-slope estimate during NREM sleep (Fig. 2A and B, lower panels) for each individual.

We implemented the algorithm described above in the Behavioural STate Analysis Toolbox (https://github.com/bnelair/best-toolbox), a Python package for behavioural state analysis using EEG. The tool was developed in the Bioelectronics Neurophysiology and Engineering Laboratory at Mayo Clinic, Rochester, MN, USA. The codes for the analysis pipeline above can be found in the following link:

https://github.com/bnelair/best-toolbox/blob/master/projects/slow_wave_detection/readme.rst.

### Statistical analysis

Numeric demographic, clinical, PSG and electrophysiological SW characteristics are described as mean ± standard deviation (SD) or median [interquartile range (IQR)], according to variable distribution. Bivariate correlations were performed preferentially with Pearson’s coefficient (*r*) with or without log-transformed variables (log), depending on variable distribution. Spearman’s rank coefficient (*rs*) was used if the linear assumption was not met or in the presence of outliers, leverage and/or influential points.

We initially used multiple linear regression models to test for linear associations between the main outcome of interest [global ΔPiB SUVR(log) or CL/year] and standard PSG measures of SWS (N3%) and OSA severity (mild versus moderate/severe). We initially adjusted associations for the most important confounders defined *a priori* based on our previous experience in a larger sample^23^: age at baseline PiB (baseline age), *APOE ɛ4* status, interval between two PiB-PET scans (PiB interval), interval between PSG and baseline PiB-PET scans (PSG-PET interval), total sleep time (TST) during the diagnostic portion of PSG and effective PAP therapy. Although the outcome variable was already annualized, we included the PiB interval in initial models to account for possible unexplained non-linearity (mostly acceleration) in accumulation at different PiB intervals, considering that most patients were in an accumulation phase at baseline before reaching deceleration (baseline global PiB 1.7 SUVR) or plateau.^50^ After inputting all variables into initial models and observing associations, *post hoc* sensitivity analyses using backward selection procedure (probability of F for removal at 0.1) were used to determine the most parsimonious models.

Additional models using SWA features instead of N3% were tested to better characterize the relationship between the different NREM SWA features and global ΔPiB(log)/year using covariates found to be most important in parsimonious models. Careful assessment for multi-collinearity was implemented by estimating the variance inflation factor to determine whether SW features could participate in the same model. Due to concern that baseline amyloid positivity (PiB+) could obscure the associations under investigation, we started the analyses without this variable and then split the analysis based on PiB status to assess how the variables of interest were associated with global ΔPiB/year in groups defined by amyloid positivity. With a reduced sample size for SWA analyses, PiB status was eventually included in the models given its importance.

*Post hoc* pairwise comparisons of SWA features between diagnostic and treatment portions of the PSG were performed using paired *t*-test followed by Cohen’s *d* effect size estimation (*d*) for paired samples (using sample SD of the mean difference adjusted by the correlation between measures) to assess for evidence of homeostatic regulation of SW features under investigation after an observation of positive associations among SO, SO-slope and amyloid accumulation.

Multiple sensitivity analyses were performed in global and regional models to test the consistency of the results, including during N3 sleep. Additional exploratory analyses investigated the relationship between global ΔPiB SUVR (log)/year and substitute variables of apnoea severity or sleep fragmentation. SPSS software for Windows version 28 (IBM Corporation) was used for statistical analyses. A two-sided *P*-value <0.05 was considered statistically significant. Due to the exploratory nature of this work, correction for multiple comparisons was not performed. MRIcroGL software^59^ was used to create neuroimaging statistical maps.

## Results

A total of 71 patients with baseline PSG and 2 consecutive PiB-PET scans were included, of which 64 had available raw EEG data with adequate signal integrity for SWA analysis. Mean (SD) age at baseline PiB was 73 ± 7.5 years old with male predominance (60.6 and 62.5%, respectively). Most patients were cognitively unimpaired at baseline PiB-PET scan (93%) and upon follow-up scan (87%). Table 1 shows full demographic, clinical and PSG characteristics. Table 2 shows SWA feature estimations.

**Table 1:** Demographic, clinical and PSG characteristics

	All (*N* = 71)	SWA analysis (*N* = 64)
Demographic characteristics		
Age at baseline PiB-PET, years, mean ± SD	72.9 ± 7.5	72.8 ± 7.7
Age at PSG, years, mean ± SD	73.3 ± 7.9	73.2 ± 8
Sex, male, *n* (%)	43 (60.6)	40 (62.5)
APOE ɛ4, any allele, *n* (%)	19 (26.8)	17 (26.6)
Education, years, mean ± SD	14.9 ± 2.5	14.7 ± 2.4
BMI at PSG, kg/m^2^, mean ± SD	31.1 ± 5.9	31.2 ± 5.9
Cognitive status		
Cognitively unimpaired at baseline PIB, *n* (%)	66 (93)	60 (93.8)
Cognitively unimpaired at follow-up PiB, *n* (%)	62 (87.3)	56 (87.5)
PiB-PET		
PSG to baseline PIB-PET, years, median (IQR)	0.7 (−0.6 to 1.7)	0.7 (−0.5 to 1.7)
Baseline PiB-PET, SUVR, median (IQR)	1.44 (1.33–1.75)	1.41 (1.32–1.70)
Baseline PiB-PET positivity, *n* (%)	31 (43.7)	25 (39.1)
Follow-up PiB-PET, SUVR, median (IQR)	1.48 (1.36–2)	1.46 (1.35–2)
Baseline PiB-PET, CL, median (IQR)	18 (8.4–45.7)	15.7 (7.5–41.8)
Follow-up PiB-PET, CL, median (IQR)	21.8 (10.7–67.7)	19.8 (10.1–70)
Interval PiB-PET scans, years, median (IQR)	2.7 (2.3–3.9)	2.7 (2.3–4)
Global ΔPiB, SUVR/year, mean ± SD	0.039 ± 0.060	0.038 ± 0.062
Global ΔPiB, SUVR(log)/year, mean ± SD	0.0085 ± 0.012	0.0081 ± 0.013
Global ΔPiB, CL/year, mean ± SD	2.1 ± 5.3	3.4 ± 5.5
OSA		
Mild, *n* (%); effective CPAP, *n* (%)	28 (39.4); 9 (32.1)	25 (39.1); 7(28)
Moderate, *n* (%); effective CPAP, *n* (%)	22 (31); 14 (63.6)	21 (32.8); 13 (62)
Severe, *n* (%); effective CPAP, *n* (%)	21 (29.6); 12 (57.1)	18 (28.1); 9 (50)
PSG parameters (diagnostic part)		
TST, min, mean ± SD	168 ± 44	168 ± 45
Sleep efficiency, %, mean ± SD	71 ± 14	71 ± 14
NREM Stage 1 (%), mean ± SD	25 ± 15	25 ± 14
NREM Stage 2 (%), mean ± SD	48 ± 14	48 ± 15
NREM Stage 3 (%), mean ± SD	15 ± 13	16 ± 13
REM stage (%), median (IQR)	11 (3–16)	11 (3–16)
Apnoea–hypopnoea index, h^−1^, median (IQR)	17 (10–31)	17 (11–31)
Respiratory disturbance index, h^−1^, median (IQR)	28 (18–38)	27.5 (18–38)
Mean SpO_2_, mean ± SD	93 ± 1.6	93 ± 1.6
Nadir SpO_2_, mean ± SD	84 ± 4.4	84 ± 4.6
Time SpO_2_ < 90%, % of TST, median (IQR)	4 (1.3–8.2)	4.4 (1.3–10.2)
Arousal index, h^−1^, median (IQR)	41.1 (30.3–53.9)	41.4 (30.5–53.3)

BMI, body mass index; global ΔPiB SUVR/year, difference between the second and first PiB-PET uptake in SUVR averaged from most susceptible regions, divided by the interval between them in years; ΔPiB SUVR(log)/year, difference between log-transformed PiB-PET SUVRs averaged from most susceptible regions, divided by the interval between them in years; REM, rapid eye movement sleep.

**Table 2:** Electrophysiological SWA parameters and comparison between diagnostic and PAP trial portions of PSG study

	SWA analysis (diagnostic) (*n* = 64)	Split-night PSG with single PAP trial (*n* = 52)		*P*	*d*
SWA parameters		Diagnostic	PAP trial		
NREM SO, relative PSD (%), mean ± SD	25.7 ± 6.6	25.7 ± 7.1	24.1 ± 6.1	0.005	0.41
NREM delta, relative PSD (%), mean ± SD	46.3 ± 5.4	46.1 ± 4.9	45.5 ± 4.5	0.240	0.17
NREM SO-slope, µV/s, mean ± SD	95.1 ± 28.9	96.4 ± 30.6	84.0 ± 23.5	<0.001	0.61
NREM delta-slope, µV/s, mean ± SD	130.8 ± 34.8	131.9 ± 36.0	115.7 ± 24.6	<0.001	0.67

### OSA severity, N3% and SWA features

AHI (log) was found to be inversely correlated with N3% (*r* = −0.30, *P* = 0.011) and delta-slope (*r* = −0.35, *P* = 0.005), but not with mean SO% (*r* = 0.07, *P* = 0.581), mean delta% (*r* = −0.23, *P* = 0.066) or SO-slope (*r* = −0.19, *P* = 0.134). Mean SpO_2_ or %time SpO_2_ below 90% were not associated with these sleep features (*P* > 0.05).

### N3% and global amyloid accumulation

**Figure 3:**
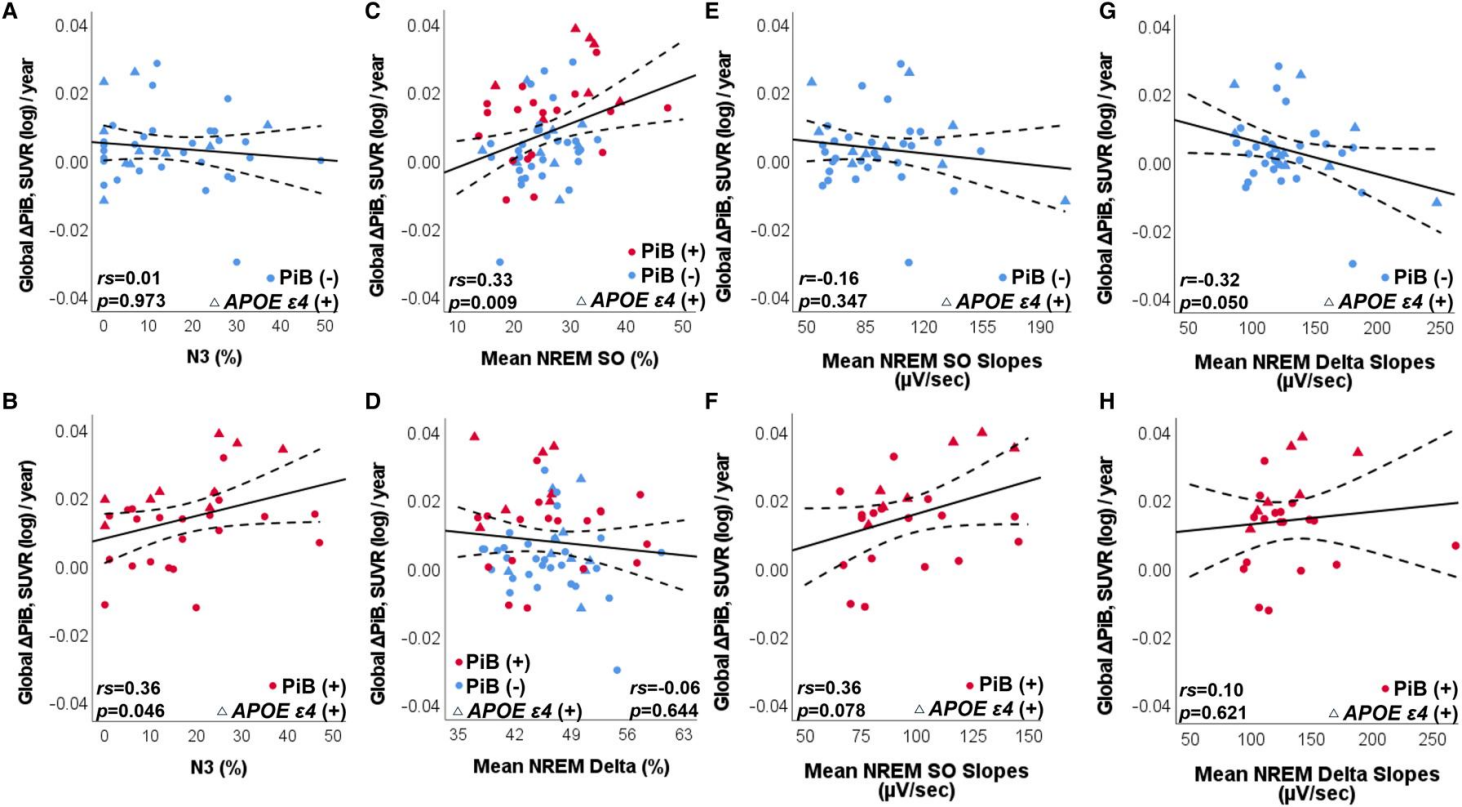
**Associations among annualized PiB-PET accumulation, SWS (N3%) and SWA features.** Unadjusted associations between global ΔPiB SUVR (log)/year and NREM Stage 3 (N3%) **(A** and **B)**, mean relative power spectral density (contribution) of SO (0.5–0.9 Hz) during NREM sleep (%) **(C**), mean relative power spectral density (contribution) of delta activity (1–3.9 Hz) during NREM sleep (**D**), mean SW slope in SO in NREM sleep **(E** and **F)** and mean SW slope in delta activity in NREM sleep **(G** and **H)**, respectively, are demonstrated according to baseline amyloid status (red = PiB+, blue = PiB−) and *APOE ɛ4* genotype-positive status (triangular shape). Best-fit lines are shown with 95% CI of mean (dashed lines). Correlation coefficient (*r* = Pearson’s and *rs* = Spearman’s) is provided for each fit line. Global ΔPiB SUVR(log)/year, difference between the second and first log-transformed PiB uptake in standardized unit value ratio (averaged across PiB-susceptible regions), divided by the interval between them in years.

We first fit linear models to assess whether global ΔPiB SUVR(log)/year was associated with N3% or OSA severity (mild versus moderate/severe), after adjusting for baseline age, PiB interval, PSG-PiB interval, *APOE ɛ4*, TST and effective PAP therapy. For every 1-SD increase in N3% between participants, there was a 0.0030 (95% CI: 0.0001; 0.0059, *P* = 0.043) increase in global ΔPiB SUVR(log)/year or 1.46 (95% CI: 0.24; 2.68, *P* = 0.019) in CL/year, which would be equivalent to 6 additional years of age at baseline (Supplementary Tables 1 and 2). OSA severity category or PAP efficacy was not significantly associated with amyloid accumulation (Supplementary Tables 1 and 2). The association with N3% persisted after the backward elimination procedure (parsimonious models; Supplementary Tables 1 and 2). By splitting the analysis by abnormal PiB at baseline, N3% remained positively associated with annualized global ΔPiB SUVR(log) or CL with greater accumulation in patients with amyloid positivity, but an association was not observed in PiB− patients (Fig. 3A and B, Table 3).

**Table 3:** Multiple linear regression model estimates for associations between standardized N3% or mean NREM SWA features and annualized global [ΔPiB SUVR(log)/year and CL/year] or regional [ΔPiB SUVR(log)/year] amyloid accumulation

	N3%^a^		Mean NREM SWA features^b^			
	PIB (+)	PiB (−)	SO%^c^	Delta%^c^	SO-slope^d^	Delta-slope^d^
ΔPiB CL/year						
Global (*β*)	2.35	−0.30	1.36	0.17	2.97	−3.17
95% CI	0.45; 4.26	−1.37; 0.76	0.09; 2.63	−1.15; 1.49	0.54; 5.39	−5.64; −0.70
*P*	**0.017**	0.57	**0.037**	0.802	**0.018**	**0.013**
Adj. *R*^2^	0.43	−0.04	0.37	0.37	0.38	0.38
ΔPiB SUVR(log)/year						
Global (*β*)	0.0044	−0.0010	0.0033	0.0003	0.0069	−0.0082
95% CI	0.0005; 0.0083	−0.0047; 0.0026	0.0001; 0.0064	−0.0030; 0.0036	0.0009; 0.0129	−0.0143; −0.0021
*P*	**0.030**	0.58	**0.042**	0.868	**0.026**	**0.009**
Adj. *R*^2^	0.37	−0.05	0.26	0.26	0.29	0.29
Orbitofrontal (*β*)	0.0064	−0.0012	0.0035	0.0007	0.0044	−0.0048
95% CI	0.0017; 0.0111	−0.0042; 0.0019	0.0002; 0.0067	−0.0027; 0.0041	−0.0020; 0.0109	−0.0114; 0.0017
*P*	**0.010**	0.439	**0.036**	0.669	0.175	0.146
Adj. *R*^2^	0.46	−0.016	0.37	0.37	0.34	0.34
Pre-frontal (*β*)	0.0057	−0.0022	0.0037	−0.0006	0.0078	−0.0091
95% CI	0.0017; 0.0098	−0.0060; 0.0016	0.0003; 0.0071	−0.0041; 0.0030	0.0012; 0.0143	−0.0158; −0.0024
*P*	**0.007**	0.255	**0.035**	0.740	**0.021**	**0.008**
Adj. *R*^2^	0.45	−0.03	0.26	0.26	0.27	0.27
Anterior and mid-cingulate (*β*)	0.0054;	−0.0019	0.0040	0.0020	0.0084	−0.0101
95% CI	−0.0001; 0.0108	−0.0061; 0.0022	0.0001; 0.0079	−0.0043; 0.0039	0.0009; 0.0159	−0.0177; −0.0025
*P*	0.054	0.35	**0.043**	0.917	**0.029**	**0.010**
Adj. *R*^2^	0.32	−0.05	0.18	0.18	0.20	0.20
Posterior cingulate/						
precuneus (*β*)	0.0025	−0.0005	0.0032	0.001102	0.0071	−0.0087
95% CI	−0.0020; 0.0070	−0.0042; 0.0032	−0.000003; 0.006	−0.0022; 0.0044	0.0011; 0.0132	−0.0148; −0.00247
*P*	0.264	0.768	40.050	0.512	**0.022**	**0.007**
Adj. *R*^2^	0.13	0.03	0.26	0.26	0.31	0.31
Parietal (*β*)	0.0027	0.0002	0.0034	0.0010	0.0079	−0.0091
95% CI	−0.0018; 0.0072	−0.0042; 0.0046	−0.0003; 0.0070	−0.0028; 0.0049	0.0011; 0.0148	−0.0161; −0.0022
*P*	0.229	0.925	0.067	0.593	**0.024**	**0.011**
Adj. *R*^2^	0.19	−0.04	0.21	0.21	0.25	0.25
Temporal (*β*)	0.0040	−0.0003	0.0027	0.0006	0.0052	−0.0064
95% CI	0.0002; 0.0077	−0.0037; 0.0031	−0.0003; 0.0058	−0.0025; 0.0038	−0.0005; 0.0110	−0.0123; −0.0006
*P*	**0.038**	0.852	0.072	0.680	0.075	**0.032**
Adj. *R*^2^	0.37	−0.05	0.25	0.25	0.27	0.27

Significant associations are marked in bold.

ΔPiB SUVR(log)/year, difference between the second and first log-transformed PiB-PET uptake in SUVR, divided by the interval between them in years; SO%, mean relative power spectral density in SO frequency range (0.5–0.9 Hz) during NREM sleep; delta%, mean relative power spectral density in delta frequency range (1–3.9 Hz) during NREM sleep; SO-slope, downslopes of SWs in SO (0.5–0.9 Hz) activity; delta-slope, downslopes of SWs in delta (1–3.9 Hz) activity.

^a^Models adjusted for baseline age and *APOE ɛ4* status.

^b^Models adjusted for baseline age, *APOE ɛ4* status and baseline PiB status.

^c^SO% and delta% participate in the same models.

^d^SO-slope and delta-slope participate in the same models.

**Figure 4:**
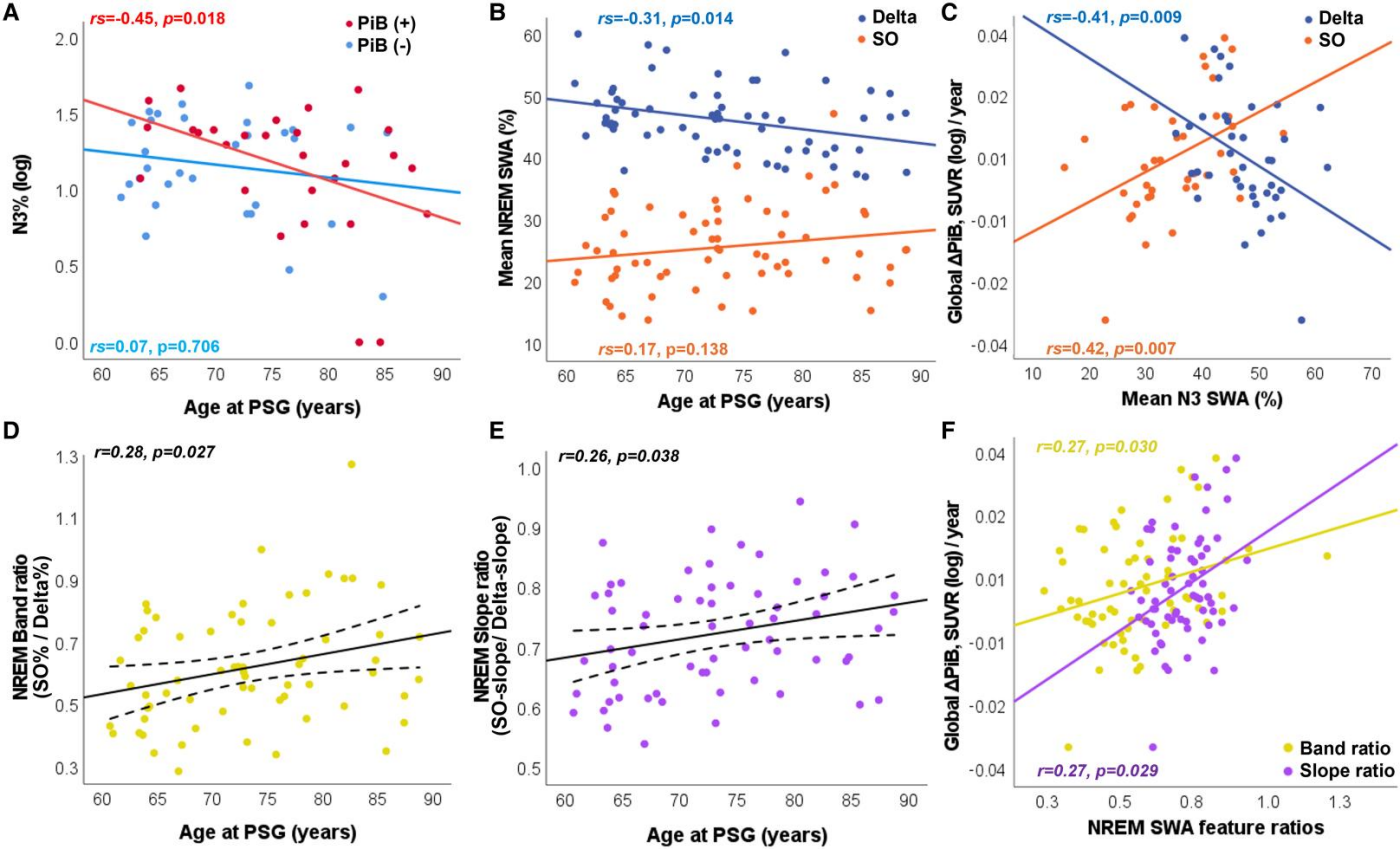
**Associations among SWS (N3%), SWA features, age at PSG study and annualized amyloid PiB-PET accumulation.** (**A**) Unadjusted associations between age at PSG and log-transformed N3% according to baseline amyloid status (red = PiB+, blue = PiB−) are demonstrated with best-fit lines based on their respective amyloid status (red line = PiB+, blue line = PiB−). (**B**) Unadjusted associations between age at PSG and the mean relative power spectral density of SWA (%) during NREM sleep as measured by the contribution of SO (0.5–0.9 Hz; orange) and delta (1–3.9 Hz; dark blue) frequency ranges are demonstrated with best fit lines for each frequency band (0.5–0.9 Hz: orange; 1–3.9 Hz: dark blue). (**C**) Unadjusted associations between annualized amyloid accumulation, as measured by global ΔPiB SUVR (log)/year, and the mean relative power spectral density of SWA (%) during N3 sleep as measured by the contribution of SO (0.5–0.9 Hz; orange) and delta (1–3.9 Hz; dark blue) frequency ranges are demonstrated with best fit lines for each frequency band (0.5–0.9 Hz: orange; 1–3.9 Hz: dark blue). Unadjusted association between age at PSG and ratio between the relative power spectral density (%) of SO and delta frequency bands during NREM sleep (SO%/delta%) (**D**) or ratio between SO-slopes and delta-slopes (SO-slope/delta-slope) during NREM sleep (**E**) are demonstrated with best fit line and 95% CI of the mean (dashed lines), respectively. Unadjusted associations between mean NREM frequency band contribution ratio (SO%/delta%, yellow colour) or mean NREM SW slope ratio (SO-slope/delta-slope, magenta colour) and global ΔPiB SUVR (log)/year are demonstrated (**F**) with best-fit line, respectively. Correlation coefficient (*r* = Pearson’s and *rs* = Spearman’s) is provided for each fit line with its colour matching the fit line colour. Global ΔPiB SUVR(log)/year, difference between the second and first log-transformed PiB uptake in standardized unit value ratio (averaged across PiB-susceptible regions), divided by the interval between them in years.

We found that age at PSG was inversely associated with N3% (log) (*r* = −0.296, *P* = 0.023), but this association was only significant in PiB+ patients (*rs* = −0.45, *P* = 0.018; Fig. 4A). A higher intercept in PiB+ patients illustrates how N3% can be positively associated with amyloid accumulation, and at the same time it is inversely associated with age (Fig. 4A).

We performed multiple sensitivity analyses to assess the consistency of the results and explore further associations. Replacing the OSA severity category with AHI (log), mean oxyhaemoglobin saturation (mean SpO_2_) or %time with SpO_2_ ≥ 90% (log) did not reveal additional associations (Supplementary Table 3). The association between N3% (log) and global ΔPiB(log)/year persisted with similar effect sizes in parsimonious models adjusted for mean SpO2 or %time with SpO_2_ ≥ 90% (log), but not AHI (log) (Supplementary Table 3), though the association between N3% and global ΔPiB(log)/year re-emerged after backward selection procedure (equal to Supplementary Table 1, model 2). Replacing N3% with N1%, sleep efficiency or arousal index did not reveal new associations (Supplementary Table 4).

### Slow-wave frequency bands and global amyloid accumulation

To investigate the components of SWA, we replaced N3% with mean SO% and delta% during NREM sleep. Due to the reduced sample size (*n* = 64), we adjusted the associations for the same covariates found to be important in the parsimonious models in both SUVR and CL units, which were baseline age and *APOE ɛ4*, and then included baseline PiB status as a covariate. We found that for every 1-SD increase in the mean SO%, there was a 0.0033 (95% CI: 0.0001; 0.0064, *P* = 0.042) increase in global ΔPiB(log)/year (Fig. 3C, Table 3), or 1.36 CL/year (95% CI: 0.09; 2.63, *P* = 0.037). This effect size was comparable to 32% of the effect size associated with baseline amyloid positivity, the strongest predictor of global ΔPiB/year (Supplementary Table 5).

Although mean delta% was not associated with global ΔPiB(log)/year (Fig. 3D, Supplementary Table 5), it was inversely associated with SO% (*r* = −0.45, *P* < 0.001). This relationship was independent of baseline global amyloid or PiB status. Mean delta% was inversely associated with age at PSG (*rs* = −0.31, *P* = 0.014), while mean SO% was not (*rs* = 0.17, *P* = 0.138; Fig. 4B). However, age at PSG was positively associated with the ratio between SWA frequencies bands (SO%/delta%; *r* = 0.28, *P* = 0.027; Fig. 4D). SWA frequency band ratio was also positively associated with global ΔPiB SUVR(log)/year (Fig. 4F, Supplementary Table 5).

For easier comparison with literature assessing N3 sleep specifically,^33,34,60^ we performed sensitivity analyses assessing for associations between mean SO%, mean delta% and global ΔPiB(log)/year during N3 sleep only in patients with at least 15 min of representation of this stage in the diagnostic portion of the study (*n* = 39). Due to the high correlation between mean SO% and delta% during N3 sleep (*r* = −0.91, *P* < 0.001), leading to collinearity concerns, we analysed these SWA features in different models. We found stronger associations between mean SO% and global ΔPiB SUVR(log)/year than that observed during NREM sleep [*β*= 0.0047 (95% CI: 0.0007; 0.0088), *P* = 0.024], after adjusting for baseline age, *APOE ɛ4*, and PiB status. Mean delta% was inversely associated with global ΔPiB(log)/year [*β*= −0.0047 (95% CI: −0.0087; −0.0007), *P* = 0.023] after adjusting for the same confounders (Fig. 4C, Supplementary Table 6).

### Slow-wave slopes and global amyloid accumulation

We also assessed whether global ΔPiB SUVR(log)/year was associated with the SW slopes in the SO (SO-slope) and delta (delta-slope) frequency bands, respectively, after adjusting for baseline age, *APOE ɛ4*, and baseline PiB positivity. For every 1-SD increase in mean SO-slope, global ΔPiB(log)/year increased by 0.0069 (95% CI: 0.0009; 0.0129, *P* = 0.026) or 2.97 CL/year (95% CI: 0.54; 5.39, *P* = 0.018); while the same increase in mean delta-slope was associated with a 0.0082 (95% CI: −0.0143; −0.0021, *P* = 0.009) reduction in global ΔPiB SUVR(log)/year (Table 3, Fig. 3E–H) or −3.17 CL/year (95% CI: −5.64; −0.70, *P* = 0.013). A 1-SD increase in SO-slope and a 1-SD reduction in delta-slope were comparable to nearly 59% and 70% of the effect size associated with baseline amyloid positivity, respectively (Supplementary Table 7). Although the mean SO-slope and delta-slope were highly correlated (*r* = 0.88, *P* < 0.001), multi-collinearity was not observed (variance inflation factor ≤5). Replacing these features by their ratio (SO-slope/delta-slope) also resulted in a positive association with global ΔPiB SUVR(log)/year (Supplementary Table 7, Fig. 4F). Although SO-slope (*r* = −0.09, *P* = 0.507) and delta-slope (*r* = −0.24, *P* = 0.055) were not associated with age at PSG, their ratio (SO-slope/delta-slope) was (*r* = 0.26, *P* = 0.038; Fig. 4E). Sensitivity analyses in patients with at least 15 min of N3 sleep (*n* = 39) required separation of these SW features in different models due to collinearity concerns. The models with SW slopes failed to show an association with global ΔPiB SUVR(log)/year in N3 sleep, but models using the slope ratio remained positively associated with global ΔPiB SUVR(log)/year (Supplementary Table 8).

### SWA features in early (diagnostic) versus late (PAP trial) sleep

To assess whether SW features investigated here display patterns of homeostatic regulation through the course of sleep, we also compared the mean SW features during NREM sleep between diagnostic and titration portion of the PSG with effect size estimations (*d*) in patients who had a titration portion with single PAP modality (continuous positive airway pressure [CPAP]; *n* = 52). Mean SO% dropped by 1.63 (95% CI: 0.51; 2.75, *P* = 0.005, *d* = 0.41), while mean delta% reduction did not reach statistical significance [0.56 (95% CI: −0.39; 1.52), *P* = 0.240]. Mean SO-slope reduced by 12.5 µV/s (95% CI: 6.76; 18.2, *P* < 0.001, *d* = 0.61) and mean delta-slope reduced by 16.2 µV/s (95% CI: 9.5; 22.9, *P* < 0.001, *d* = 0.67; Table 2). The larger the reduction in AHI from the diagnostic to PAP trial portion, the smaller the reduction in mean SO% (*rs* = −0.35, *P* = 0.012), SO-slopes (*rs* = −0.42, *P* = 0.002) and delta-slopes (*rs* = −0.5, *P* < 0.001). Mean delta% difference was not associated with AHI reduction (*rs* = −0.05, *P* = 0.702; Supplementary Fig. 1). However, for all SWA metrics, the greater their means at baseline, the greater their reduction. The lower the mean SW feature, the lower the reduction, which in some patients represented an increase in the mean of one or more SW features during the PAP trial (Supplementary Fig. 2).

### SWA features and regional amyloid accumulation

Given that SWA measured here are frontally predominant (Fz), we first assessed whether baseline pre-frontal or orbitofrontal PiB-PET SUVR was associated with (i) mean SO% and delta% or (ii) mean SO-slope and delta-slope, respectively, while adjusting for baseline age, *APOE ɛ4*, PSG-PiB interval and OSA severity (mild versus moderate/severe). Standardized mean SO% was associated with baseline log-transformed PiB in the pre-frontal [*β*= 0.0282 (95% CI: 0.0039; 0.0525), *P* = 0.024] and orbitofrontal regions [*β*= 0.0280 (95% CI: 0.0010; 0.0544), *P* = 0.042], while delta activity%, SW slopes and OSA severity were not.

**Figure 5:**
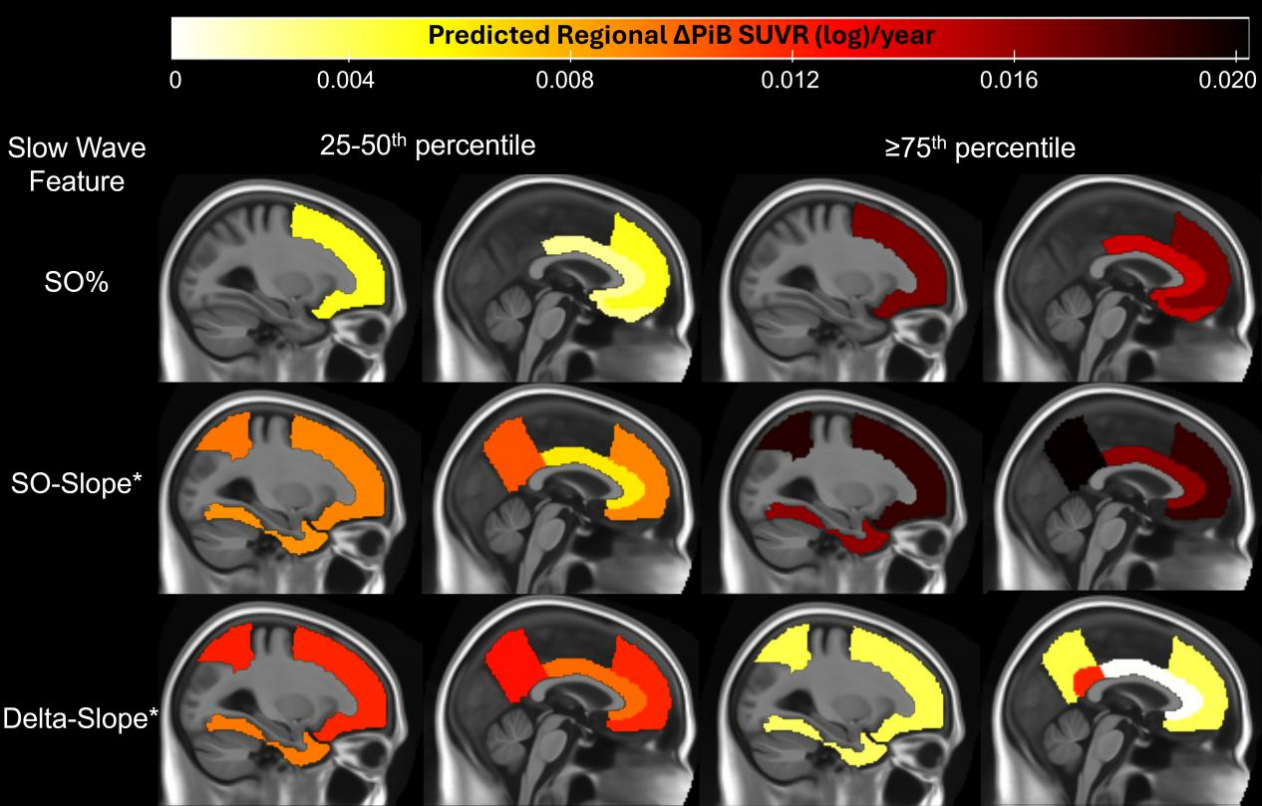
**Predicted regional annualized PiB-PET accumulation according to NREM slow-wave features.** Comparison of predicted mean regional ΔPiB SUVR(log)/year estimates for patients in the second (25–50th percentiles) and fourth (≥75th percentile) quartiles of each slow-wave feature distribution in regions with significant associations, after adjusting for baseline age, *APOE ɛ4* and baseline amyloid positivity. *Given that SO-slope and delta-slope participate in the same models, comparisons for each feature (e.g. SO-slope) are demonstrated only for patients in the same quartile (50–75th percentiles) of the other feature (e.g. Delta-slope). ΔPiB(log)/year, difference between the second and first log-transformed PiB uptake in SUVR), divided by the interval between them in years; SO%, mean SO (0.5–0.9 Hz) contribution (%) as measured by its relative spectral power density during NREM sleep; SO-slope, mean SW slope in SOs during NREM sleep; delta-slope, mean SW slope in delta activity (1–3.9 Hz) during NREM sleep.

Second, we tested for associations between regional amyloid accumulation measured by ΔPiB SUVR(log)/year in each ROI and (i) mean SO% and mean delta% and (ii) mean SO-slope and delta-slope, respectively. We adjusted the associations for covariates found to be most important in global models for both SUVR/year and CL/year: age at baseline, *APOE ɛ4* and baseline amyloid positivity. Models with N3% split by PiB status were included for comparison. We found stronger associations in pre-frontal regions, which were associated positively with mean SO% and SO-slope and negatively with delta-slope (Table 3). Models with slopes had higher effect sizes and more widespread regional associations (Table 3, Fig. 5).

## Discussion

We found that longitudinal global and regional annualized log-transformed amyloid accumulation measured by PiB-PET in predominantly cognitively unimpaired OSA patients was positively associated with the contribution of N3%, mean SO% and mean SO-slope. Mean delta-slope was associated with reduced PiB-PET accumulation. Sleep apnoea severity was not associated with PiB-PET accumulation. Although our findings were contrary to our initial assumptions, we hypothesize that OSA and/or underlying Alzheimer’s disease pathology lead to altered sleep homeostasis, possibly due to unmet normalization of synaptic strength,^61,62^ and hyper-excitability^38^ before the onset of cognitive decline.

Most of what is known about the relationship between SWA and amyloid burden or accumulation is based on seminal work from the same group of investigators studying fundamentally different samples and using different methodologies.^33,34,63^ They showed that reduced SO (0.6–1 Hz) at baseline is associated with greater cross-sectional pre-frontal^33^ and global amyloid burden^63^ and greater longitudinal global Aβ accumulation in healthy older adults,^34^ in contrast to our findings. However, their sample probably had a lower amyloid burden at baseline when compared with ours (taking into consideration different PiB+ thresholds) and much lower sleep apnoea and self-reported sleep disturbance burden.^34^ They studied SWA features specifically during N3 sleep throughout the night, while we studied them during NREM/N3 sleep in the first hours of sleep. This methodological difference is relevant because homeostatic pressure for sleep is mostly reflected in SWA,^64-69^ with higher contribution, amplitudes and slopes at the beginning of sleep,^39,70^ with decay during the course of sleep. Our findings of smaller reductions (or even increases) in mean SO% and mean SW slopes from diagnostic to PAP trial portions in patients with greater reduction in apnoea burden upon treatment are in agreement with findings of increased homeostatic sleep pressure in OSA due to unmet sleep needs with rebound increases in SWS upon treatment^71,72^ and slower exponential decay rate of SWA during sleep.^72,73^ Some authors found increases in SWA% or slowing (delta/alpha ratio) in OSA patients, despite reduced N3 sleep.^74,75^ Increased SWA has been associated with increased AHI, hypoxaemia measures, arousal index and hypercapnia,^74,76^ though associations between OSA severity parameters and SWA have been inconsistent.^74,75,77-79^

Therefore, it is possible that both reductions in SO (across all N3, in the absence of OSA/increased homeostatic sleep pressure) and increases in SO (early in NREM/N3 sleep, in the setting of OSA/increased homeostatic sleep pressure) could be related to amyloid accumulation. The notion of a physiological ceiling of SO and SWA is corroborated by acoustic SO enhancement studies indicating that SO increases in ON intervals are counteracted by a reduction in OFF intervals, resulting in an absence of overall change in SWS contribution.^80,81^ Therefore, an increase in SO/SWA in OSA may be adaptive/compensatory or pathological.

In another study of healthy elderly with low amyloid burden at baseline, neither SWA band was associated with amyloid accumulation.^82^ However, in older adults with higher amyloid burden, mean cortical amyloid burden was associated with reduction in delta (1–2 Hz) during NREM sleep cross-sectionally, but SO was not studied.^25^ Given the inverse relationship between SO and delta contribution described herein, it may be speculated that the reduction in delta might have been associated with an actual increase in SO, similar to our findings. In healthy older adults, SWS duration, SWS% and frontal 0.5–4 Hz SWA (in the first two NREM cycles) were found to be negatively associated with CSF Aβ42 levels.^29^ Although experimental acute reduction/disruption of SWA/SWS promotes higher CSF Aβ levels at early stages,^83^ CSF Aβ42 levels decline in older adults.^84^ Therefore, lower CSF Aβ42 levels with increased SWS/SWA in these participants may actually represent more amyloid aggregation, in agreement with our results, and consistent with evidence suggesting decreased CSF Aβ42 with higher OSA severity^18,19^ and worse hypoxaemia.^12^ Some longitudinal studies have shown associations between subjectively reported OSA or objectively measured OSA severity and decreased CSF Aβ42^15,19^ and greater amyloid PET accumulation,^15^ but SWA was not assessed.

Altered sleep homeostasis in OSA may reflect increased synaptic strength and hyper-excitability due to disrupted sleep. Multiple studies support a net increase in synaptic strength and excitability during wakefulness, which is reduced (down-scaled) during sleep^85-93^ to prevent an increase in energy utilization, hyper-excitability and runaway potentiation. Increased SWA has been implicated in increased synaptic potentiation/strength^85,94,95^ and learning.^69,96-98^ Homeostatic sleep pressure has been linked primarily to delta and SW slopes,^57,70,99^ but SO has also been implicated.^100^ Still, our findings of stronger associations between SW slopes with PiB-PET accumulation than SO% and larger reductions through the course of sleep are consistent with previous findings suggesting that SW slopes may be more sensitive markers of sleep homeostasis due to (i) steeper decline throughout the night^39,70^ and (ii) steeper slope following sleep deprivation.^57^

Homeostatic regulation of SWA can also be influenced by ageing,^101-105^ with less contribution and overnight attenuation of delta during sleep,^58,101,102,105^ in agreement with our findings of decreased mean delta% with aging. However, our results also suggest an increase in SO contribution with ageing (Fig. 3B–C), consistent with a large-scale microstructural sleep EEG study,^58^ which also showed that higher relative contribution of SO and less contribution of the delta were associated with worse cognitive performance,^58^ supporting the importance of a fine balance between both, as suggested by other authors.^106^ A higher relative contribution of SO may be consistent with findings of longer SW positive and negative phases with ageing.^107^ SW slopes also appear to reduce with ageing.^36,107,108^ However, in CU older adults, higher contribution of faster negative-to-positive peak transitions in SWs or ‘fast switchers’ (suggesting higher slopes)^109^ was inversely associated with cognitive performance.^110^

Although SWA is thought to have a predominant local/regional origin,^111^ high-amplitude SWs are usually a global phenomenon in early NREM sleep.^111^ Moreover, transitions from wake to sleep may give rise to SWA with two distinct network synchronization processes.^112^ In the setting of increased sleep fragmentation and arousals from ageing and OSA, increased SO and SO-slopes may reflect an increase in ‘Type 1’ (long-duration, steeper slope) SWs, which may have a wider synchronization pattern (subcortical–cortical) and predominate early upon falling back asleep, as opposed to ‘Type 2’ (short-duration, smaller slope) SWs, driven by local cortico–cortico connections, which predominate later.^112^ Accordingly, N1 duration was positively associated with SWA band difference (SO%−delta%; *rs* = 0.347, *P* = 0.005). A wider synchronization pattern may be more resilient to age-related regional cortical thinning in areas involved with SWA generation and propagation^60,113^ than local connections.

Changes in SWA and SW slopes may also reflect excitatory–inhibitory imbalance,^38,114-116^ which may be related to both OSA and Alzheimer’s disease pathology.^117^ Sleep fragmentation and chronic intermittent hypoxia may increase neuronal activity and cause hyper-excitability,^118-121^ which can promote Aβ42 production.^122-125^ Dysregulation of Aβ42 and its precursor in homeostatic synaptic plasticity may further contribute to hyper-excitability early in the Alzheimer’s disease process, before hypoactivity occurs.^126-133^ Interestingly, healthy young men with higher risk for Alzheimer’s disease based on genome-wide polygenic risk scores were found to have higher SWS contribution in both SO (0.5–1 Hz) and delta (1.25–4 Hz) frequency ranges during habitual sleep and more overall SWA following sleep loss, possibly due to increased neuronal activity even before amyloid accumulation would be expected.^134^

In older adults with OSA, greater amyloid burden in the posterior cingulate/precuneus region was observed and was related to greater grey matter volume, perfusion and metabolism over the same regions,^17^ consistent with other studies describing increased CSF lactate,^12^ decreases in gamma-aminobutyric acid (GABA),^135,136^ increases in glutamate,^136^ cortical hypertrophy^137,138^ and hypermetabolism^139,140^ in patients with OSA, especially in regions linked to SWA generation and propagation.^141,142^ These findings may reflect early changes related to increased neuronal activity not captured by other studies.^143,144^ Previous experimental work has shown that the higher the firing rate during SW up states upon decreased inhibition, the longer the SW down states and the higher the slopes.^38^ Disinhibition also facilitated a more synchronized network.^38^ All of these changes could be consistent with increased SWS, SO and SW slopes.

Last, we did not find an association between OSA severity and PiB-PET measures. This bears similarity to another study in which apnoea severity was not associated with the annual change in PiB-PET signal, though found that apnoea severity was significantly associated with an annual decrease in CSF Aβ42.^19^ However, it differs from observations suggesting CU individuals with OSA have a higher amyloid PET burden than individuals without OSA, with 8% of the variance explained by a composite hypoxaemia measure.^17^ In individuals with mild cognitive impairment, higher AHI was also associated with a higher global amyloid PET burden.^20^ Our observations here may be influenced by a mixture of participants who did or did not undergo effective therapy for OSA. Other mitigating factors may be related to limitations of standard PSG metrics for OSA severity,^145^ night-to-night variability of respiratory events,^146^ different OSA endophenotypes^147,148^ and arousal thresholds.^149^ Our ability to detect associations related to SWA changes and not to OSA severity metrics may also suggest that SWA changes are a downstream process, reflecting different susceptibility to sleep disturbance.

The study has several limitations. Despite participation in the MCSA, this is ultimately a convenience clinical sample that underwent PSG for sleep concerns, which may limit the generalizability of findings to the community. Conversely, participants of the MCSA with OSA are more likely to have better longitudinal care and may be healthier than other patients with OSA from the community or other potentially underserved areas. Although this retrospective analysis is longitudinal, Alzheimer’s disease pathology is thought to arise much earlier than detectable by PiB-PET scans,^11^ preventing the determination of cause–effect relationships.

The diagnostic portion of an in-lab split-night PSG study is not representative of a full night of sleep and is subject to the first-night effect. However, SWS is maximal at its first period with a declining trend through the night,^66^ which may make this portion suitable for studying early SWS/SWA features.^107,113^ Moreover, a meta-analysis showed that the SWS representation is not significantly affected by the first-night effect.^150^ Although EEG SWA in early NREM sleep originates predominantly in frontal derivations (including Fz)^151^ and generally propagates in anteroposterior direction in the brain,^111,151^ often involving all the ROIs studied here,^142^ the lack of multi-channel EEG data precluded an analysis assessing global versus local (non-global) SWs and their propagation, which might have obscured some associations between some SWA features and regional amyloid accumulation further away from frontal regions. The lack of longitudinal sleep assessments also precluded the identification of SWA features following OSA treatment, with possible normalization of increased homeostatic pressure; hence, baseline SWA features may not reflect SWA characteristics during most of the PiB interval and should be interpreted with caution.

Due to the retrospective nature of the study, we cannot exclude the effects of acute/chronic sleep restriction.^152^ Our study was likely under-sampled to assess associations among OSA severity, CPAP efficacy and amyloid accumulation. Finally, it is important to recognize that there is much more to OSA than amyloid pathology,^8,10,22,153^ which may also be related to SWA changes^153^ and cognitive decline. OSA may also interact with other comorbidities, which may synergistically contribute to amyloid accumulation.^154^ Although important, these investigations were beyond the scope of the present work.

## Conclusion

Our study illustrates the complexity of SWA in the context of OSA, ageing and Alzheimer’s disease pathology. Overall, the results support that OSA is a state of increased homeostatic pressure with increased synaptic strength and hyper-excitability, further exacerbated by possible underlying early Alzheimer’s disease pathology. In the setting of ageing-related decreases in the homeostatic regulation of delta and more sleep disturbance, the SO contribution may increase with higher SW slopes in connection with amyloid accumulation at early stages. Despite reduced expression of delta with ageing, its slopes may still signal residual homeostatic regulation and may therefore represent protection against amyloid pathology. Further studies will be required to better understand the relationship between SW features and Alzheimer’s disease process in ageing and sleep apnoea longitudinally with multi-modal imaging. A more comprehensive understanding of SWA dynamics in the presence of sleep disorders (before and after treatment) is necessary for the development of sleep-based interventions to restore physiological brain activity.

## Supplementary Material

fcae354_Supplementary_Data

## Data Availability

Anonymized data will be available upon reasonable request from a qualified investigator in accordance with the Mayo Clinic and MCSA data-sharing protocols.
