# Supplementary material for: Non-rapid eye movement sleep slow-wave activity features are associated with amyloid accumulation in older adults with obstructive sleep apnoea
Source: Brain Commun. 2024 Oct 7;6(5):fcae354. doi: 10.1093/braincomms/fcae354 (PMC11487750; doi:10.1093/braincomms/fcae354)
Supplement: fcae354_Supplementary_Data [file fcae354_supplementary_data.docx]

Supplementary table 1. Multiple linear regression model estimates for associations between standardized N3% and annualized global amyloid accumulation (ΔPiB SUVR[log]/year).

| Models | Variables (Predictors) | *β* (95% CI) | *P* |
| --- | --- | --- | --- |
| 1 – Initial | **N3% (z-scored)** | **0.0030 (0.0001; 0.0059)** | **0.043** |
|  | Moderate/Severe OSA (vs. mild OSA) | -0.0051(-0.0114; 0.0012) | 0.108 |
|  | **Baseline age (years)** | **0.0005 (0.00012; 0.0009)** | **0.012** |
|  | PiB interval (years) | 0.0018 (-0.00002; 0.0036) | 0.052 |
|  | PSG-PIB interval (years) | -0.0008 (-0.0024; 0.0008) | 0.323 |
|  | **APOE ε4 (any allele vs. none)** | **0.0111 (0.0493; 0.0173)** | **<0.001** |
|  | Diagnostic TST (minutes) | -0.00001 (-0.00008; 0.00006) | 0.744 |
|  | Effective PAP therapy (yes vs. no) | 0.0015 (-0.0041; 0.0071) | 0.591 |
|  |  |  |  |
|  |  |  |  |
| 2 – Parsimonious model | **N3% (z-scored)** | **0.0029 (0.0001; 0.0058)** | **0.045** |
|  | **Baseline age (years)** | **0.0004 (0.00004; 0.0008)** | **0.032** |
|  | PiB interval (years) | 0.0015 (-0.0003; 0.0032) | 0.095 |
|  | **APOE ε4 (any allele vs. none)** | **0.0106 (0.0045; 0.0166)** | **<0.001** |
|  |  |  |  |
|  |  |  |  |
| 3 – Baseline PiB+ patients | **N3% (z-scored)** | **0.0044 (0.0004; 0.0083)** | **0.033** |
|  | Baseline age (years) | 0.0001 (-0.0005; 0.0007) | 0.709 |
|  | PiB interval (years) | -0.0005 (-0.0032; 0.0023) | 0.731 |
|  | **APOE ε4 (any allele vs. none)** | **0.0142 (0.0062; 0.0223)** | **0.001** |
|  |  |  |  |
| 4 – Baseline PiB- patients | N3% (z-scored) | 0.0002 (-0.0036; 0.0040) | 0.922 |
|  | Baseline age (years) | 0.0002 (-0.0003; 0.0007) | 0.427 |
|  | PiB interval (years) | 0.0018 (-0.0002; 0.0039) | 0.080 |
|  | APOE ε4 (any allele vs. none) | 0.0040 (-0.0041; 0.0122) | 0.323 |

Supplementary Table 1 legend. ΔPiB SUVR(log)/year: difference between the second and first log-transformed Pittsburgh B compound (PiB)-PET uptake in standardized uptake value ratio (SUVR), divided by the interval between them in years. N3: Non-REM sleep stage 3. OSA: obstructive sleep apnea. PSG: polysomnography: TST: total sleep time. PAP: positive airway pressure. Significant associations are marked in bold.

Supplementary Table 2. Multiple linear regression model estimates for associations between standardized N3% and annualized global amyloid accumulation in centiloid units (ΔPiB CL/year).

| Models | Variables (Predictors) | *β* (95% CI) | *P* |
| --- | --- | --- | --- |
| 1A - Model 1 in CL | **N3% (z-scored)** | **1.46 (0.24; 2.68)** | **0.019** |
|  | Moderate/Severe OSA (vs. mild OSA) | -1.95 (-4.60; 0.71) | 0.147 |
|  | **Baseline age (years)** | **0.23 (0.07; 0.39)** | **0.007** |
|  | PiB interval (years) | 0.50 (-0.25; 1.26) | 0.187 |
|  | PSG-PIB interval (years) | -0.58 (-1.26; 0.11) | 0.098 |
|  | **APOE ε4 (any allele vs. none)** | **5.23 (2.61; 7.85)** | **<0.001** |
|  | Diagnostic TST (minutes) | -0.001 (-0.03; 0.03) | 0.962 |
|  | Effective PAP therapy (yes vs. no) | -0.290 (-2.65; 2.07) | 0.807 |
|  |  |  |  |
|  |  |  |  |
| 2A – Parsimonious CL model | **N3% (z-scored)** | **1.33 (0.18; 2.48)** | **0.024** |
|  | **Baseline age (years)** | **0.16 (0.005; 0.31)** | **0.043** |
|  | **APOE ε4 (any allele vs. none)** | **4.79 (2.24; 7.34)** | **<0.001** |
|  |  |  |  |
|  |  |  |  |
| 3A – Baseline PiB+ patients | **N3% (z-scored)** | **2.35 (0.45; 4.26)** | **0.017** |
|  | Baseline age (years) | 0.08 (-0.18; 0.34) | 0.537 |
|  | **APOE ε4 (any allele vs. none)** | **8.04 (4.25; 11.8)** | **<0.001** |
|  |  |  |  |
| 4A – Baseline PiB- patients | N3% (z-scored) | -0.30 (-1.37; 0.76) | 0.568 |
|  | Baseline age (years) | 0.04 (-0.12; 0.19) | 0.632 |
|  | APOE ε4 (any allele vs. none) | 0.93 (-1.50; 3.35) | 0.443 |
|  |  |  |  |

Supplementary Table 2 legend. ΔPiB CL/year: difference between the second and first Pittsburgh B compound (PiB)-PET uptake in centiloid-converted standardized uptake value ratio (SUVR), divided by the interval between them in years. N3%: Percentage of non-rapid eye movement (NREM) stage 3. OSA: obstructive sleep apnea. PSG: polysomnography: TST: total sleep time. PAP: positive airway pressure. Significant associations are marked in bold.

Supplementary Table 3. Multiple linear regression model estimates for associations between N3 (%) and annualized global amyloid accumulation (ΔPiB SUVR[log]/year) for different models with substitute OSA severity measures.

| Models | Variables (Predictors) | *β* (95% CI) | *P* |
| --- | --- | --- | --- |
| 5 – AHI (log) added to | N3 (%) | 0.0002 (-0.0003; 0.0043) | 0.085 |
| Parsimonious model (2) | AHI (log) | -0.0045 (-0.0140; 0.0051) | 0.355 |
|  | Baseline age (years) | 0.0004 (-0.00002; 0.0008) | 0.065 |
|  | PiB interval (years) | 0.0011 (-0.0007; 0.0028) | 0.225 |
|  | **APOE ε4 (any allele vs. none)** | **0.0103 (0.0040; 0.0166)** | **0.002** |
|  |  |  |  |
| 6 – Mean SpO2 added to | **N3 (%)** | **0.0022 (0.00002; 0.0004)** | **0.048** |
| Parsimonious model (2) | Mean SpO2 (log) (%) | 0.0013 (-0.0005; 0.0030) | 0.147 |
|  | Baseline age (years) | 0.0003 (-0.0001; 0.0007) | 0.118 |
|  | PiB interval (years) | 0.0011 (-0.0007; 0.0028) | 0.223 |
|  | **APOE ε4 (any allele vs. no allele)** | **0.0101 (0.0039; 0.0163)** | **0.002** |
|  |  |  |  |
| 7 – Hypoxia burden added | **N3 (%)** | **0.0023 (0.00006; 0.0005)** | **0.044** |
| To Parsimonious model (2) | % Time SpO2 ≥ 90% (log) | 0.0185 (-0.012; 0.049) | 0.228 |
|  | Baseline age (years) | 0.0003 (-0.00006; 0.0007) | 0.094 |
|  | PiB interval (years) | 0.0011 (-0.0007; 0.0028) | 0.230 |
|  | **APOE ε4 (any allele vs. no allele)** | **0.0010 (0.0038; 0.0162)** | **0.002** |

Supplementary Table 3 legend. ΔPiB SUVR(log)/year: difference between the second and first log-transformed Pittsburgh B compound (PiB)-PET uptake in standardized uptake value ratio (SUVR), divided by the interval between them in years. AHI: apnea-hypopnea index. SpO2: oxyhemoglobin saturation. Significant associations are marked in bold.

Supplementary Table 4. Multiple linear regression model estimates for associations between exploratory sleep fragmentation measures (instead of N3%) and annualized global amyloid accumulation (ΔPiB SUVR[log]/year) in parsimonious model (2).

| Models | Variables (Predictors) | *β* (95% CI) | *P* |
| --- | --- | --- | --- |
| 8 – N1% | N1 (%) | -0.0001 (-0.0003; 0.0001) | 0.430 |
|  |  |  |  |
| 9 – Sleep efficiency | Sleep efficiency (%) | -0.00004 (-0.0003; 0.0002) | 0.706 |
|  |  |  |  |
| 10 – Arousal index | Arousal index (events/hour) | -0.00008 (-0.0002; 0.0001) | 0.242 |

Supplementary Table 4 legend. ΔPiB SUVR(log)/year: difference between the second and first log-transformed Pittsburgh B compound (PiB)-PET uptake in standardized uptake value ratio (SUVR), divided by the interval between them in years. N1%: percentage of non-rapid eye movement (NREM) stage 1.

Supplementary Table 5. Multiple linear regression model estimates for associations between NREM SWA features (mean SO% and mean delta%) and annualized global amyloid accumulation (ΔPiB SUVR[log]/year or CL/year).

| Models | Variables (Predictors) | *β* (95% CI) | *P* |
| --- | --- | --- | --- |
| 11 – SUVR (log)/year | **Mean SO% (z-scored)** | **0.0033 (0.0001; 0.0064)** | **0.042** |
|  | Mean delta% (z-scored) | 0.0003 (-0.0030; 0.0036) | 0.868 |
|  | Baseline age (years) | -0.00008 (-0.0005; 0.0003) | 0.700 |
|  | **APOE ε4 (any allele vs. none)** | **0.0074 (0.0011; 0.0136)** | **0.022** |
|  | **PiB status (PiB+ vs. PiB-)** | **0.0102 (0.0040; 0.0163)** | **<0.001** |
|  |  |  |  |
| 12 – SUVR (log)/year | **Mean SO%/mean delta% (ratio)** | **0.0156 (0.0002; 0.0311)** | **0.048** |
|  | Baseline age (years) | -0.0001(-0.0005; 0.0003) | 0.522 |
|  | **APOE ε4 (any allele vs. none)** | **0.0078 (0.0016; 0.0140)** | **0.015** |
|  | **PiB status (PiB+ vs. PiB-)** | **0.0104 (0.0044; 0.0165)** | **<0.001** |
|  |  |  |  |
| 11 A – CL/year | **Mean SO (%)** | **1.36 (0.09; 2.63)** | **0.037** |
|  | Mean delta (%) | 0.17 (-1.15; 1.49) | 0.802 |
|  | Baseline age (years) | -0.04 (-0.21; 0.13) | 0.651 |
|  | **APOE ε4 (any allele vs. none)** | **3.90 (1.38; 6.42)** | **0.003** |
|  | **PiB status (PiB+ vs. PiB-)** | **5.28 (2.81; 7.75)** | **<0.001** |

Supplementary Table 5 legend: NREM: non-rapid eye movement sleep stage. ΔPiB SUVR(log)/year = difference between the second and first log-transformed Pittsburgh B compound (PiB)-PET uptake in standardized uptake value ratio (SUVR), divided by the interval between them in years. CL= centiloid unit-converted SUVR. SO%: mean relative power spectral density in slow oscillation frequency range (0.5-0.9 Hz) during NREM sleep. Delta%: mean relative power spectral density in delta frequency range (1-3.9 Hz) during NREM sleep. Significant associations are marked in bold.

Supplemental Table 6. Multiple linear regression model estimates for associations between standardized N3 SWA features (mean SO% and mean delta%) and annualized global amyloid accumulation (ΔPiB SUVR[log]/year).

| Models | Variables (Predictors / Covariates) | *β* (95% CI) | *P* |
| --- | --- | --- | --- |
| 13 – N3 model | **Mean SO (%) (z-scored)** | **0.0047 (0.0007; 0.0088)** | **0.024** |
|  | Baseline age (years) | -0.0003 (-0.0008; 0.0003) | 0.331 |
|  | APOE ε4 (any allele vs. none) | 0.0065 (-0.0031; 0.0161) | 0.176 |
|  | **PiB status (PiB+ vs. PiB-)** | **0.0125 (0.0044; 0.0206)** | **0.003** |
|  |  |  |  |
| 14 – N3 model | **Mean delta (%) (z-scored)** | **-0.0047 (-0.0087; -0.0007)** | **0.023** |
|  | Baseline age (years) | -0.0003 (-0.0008; 0.0003) | 0.315 |
|  | APOE ε4 (any allele vs. none) | 0.0071 (-0.0023; 0.0165) | 0.134 |
|  | **PiB status (PiB+ vs. PiB-)** | **0.0128 (0.0047; 0.0209)** | **0.003** |

Supplementary Table 6 legend: N3: non-rapid eye movement stage 3 sleep. ΔPiB SUVR(log)/year = difference between the second and first log-transformed Pittsburgh B compound (PiB)-PET uptake in standardized uptake value ratio (SUVR), divided by the interval between them in years. CL= centiloid unit-converted SUVR. SO%: mean relative power spectral density in slow oscillation frequency range (0.5-0.9 Hz) during N3 sleep. Delta%: mean relative power spectral density in delta frequency range (1-3.9 Hz) during N3 sleep. Significant associations are marked in bold.

Supplementary Table 7. Multiple linear regression model estimates for associations between NREM SWA features (mean SO-slope and mean delta-slope) and annualized global amyloid accumulation (ΔPiB SUVR[log]/year or CL/year).

| Models | Variables (Predictors) | *β* (95% CI) | *P* |
| --- | --- | --- | --- |
| 15 – SUVR (log)/year | **SO-slope (z-scored)** | **0.0069 (0.0009; 0.0129)** | **0.026** |
|  | **Delta-slope (z-scored)** | **-0.0082 (-0.0143; -0.0021)** | **0.009** |
|  | Baseline age (years) | -0.0002 (-0.0006; 0.0002) | 0.259 |
|  | **APOE ε4 (any allele vs. none)** | **0.0077 (0.0014; 0.0139)** | **0.017** |
|  | **PiB status (PiB+ vs. PiB-)** | **0.0117 (0.0057; 0.0177)** | **<0.001** |
|  |  |  |  |
| 16 – SUVR (log)/year | **SO-slope / delta-slope (ratio)** | **0.035 (0.0047; 0.0654)** | **0.025** |
|  | Baseline age (years) | -0.000 (-0.0005; 0.0003) | 0.506 |
|  | **APOE ε4 (any allele vs. none)** | **0.0073 (0.0010; 0.0135)** | **0.023** |
|  | **PiB status (PiB+ vs. PiB-)** | **0.0105 (0.0045; 0.0165)** | **<0.001** |
|  |  |  |  |
| 15 A – CL/year | **SO-slope (z-scored)** | **2.97 (0.54; 5.39)** | **0.018** |
|  | **Delta-slope (z-scored)** | -3.17 (-5.64; -0.70) | 0.013 |
|  | Baseline age (years) | -0.09 (-0.26; 0.07) | 0.263 |
|  | **APOE ε4 (any allele vs. none)** | **3.91 (1.39; 6.43)** | **0.003** |
|  | **PiB status (PiB+ vs. PiB-)** | **5.86 (3.42; 8.29)** | **<0.001** |

Supplementary Table 7 legend: NREM: non-rapid eye movement sleep stage. ΔPiB SUVR(log)/year = difference between the second and first log-transformed Pittsburgh B compound (PiB)-PET uptake in standardized uptake value ratio (SUVR), divided by the interval between them in years. CL= centiloid unit-converted SUVR. SO-slope: downslopes of the slow waves in slow oscillation (0.5-0.9 Hz) activity during NREM sleep. Delta-slope: downslopes of the slow waves in delta (1-3.9 Hz) activity during NREM sleep. Significant associations are marked in bold.

Supplemental Table 8. Multiple linear regression model estimates for associations between N3 SWA features (mean SO-slope and mean delta-slope) and annualized global amyloid accumulation (ΔPiB SUVR[log]/year).

| Models | Variables (Predictors / Covariates) | *β* (95% CI) | *P* |
| --- | --- | --- | --- |
| 17 – N3 model | SO-slope | 0.000004 (-0.0001; 0.0001) | 0.945 |
|  | Baseline age (years) | -0.0001 (-0.0007; 0.0005) | 0.640 |
|  | APOE ε4 (any allele vs. none) | 0.0106 (0.0009; 0.0203) | 0.033 |
|  | **PiB status (PiB+ vs. PiB-)** | **0.0120 (0.0033; 0.0210)** | **0.009** |
|  |  |  |  |
| 18 – N3 model | Delta-slope | -0.0001 (-0.0001; 0.00004) | 0.232 |
|  | Baseline age (years) | -0.0001 (-0.0007; 0.0004) | 0.604 |
|  | APOE ε4 (any allele vs. none) | 0.0106 (0.0012; 0.0201) | 0.028 |
|  | **PiB status (PiB+ vs. PiB-)** | **0.0125 (0.0039; 0.0211)** | **0.005** |
|  |  |  |  |
| 19 – N3 model | **SO-slope / Delta-slope (ratio)** | **0.0754 (0.0306; 0.1201)** | **0.002** |
|  | Baseline age (years) | -0.0005 (-0.0010; 0.0001) | 0.091 |
|  | APOE ε4 (any allele vs. none) | 0.0065 (-0.0021; 0.0152) | 0.135 |
|  | **PiB status (PiB+ vs. PiB-)** | **0.0753 (0.0306; 0.1201)** | **0.002** |

Supplementary Table 8 legend: N3: non-rapid eye movement stage 3 sleep. ΔPiB SUVR(log)/year = difference between the second and first log-transformed Pittsburgh B compound (PiB)-PET uptake in standardized uptake value ratio (SUVR), divided by the interval between them in years. SO-slope: downslopes of the slow waves in slow oscillation (0.5-0.9 Hz) activity during N3 sleep. Delta-slope: downslopes of the slow waves in delta (1-3.9 Hz) activity during N3 sleep. Significant associations are marked in bold.

Supplementary Figure 1 heading: Associations between differences in NREM SWA features and apnea severity indices between diagnostic and PAP trial portions of the sleep study.


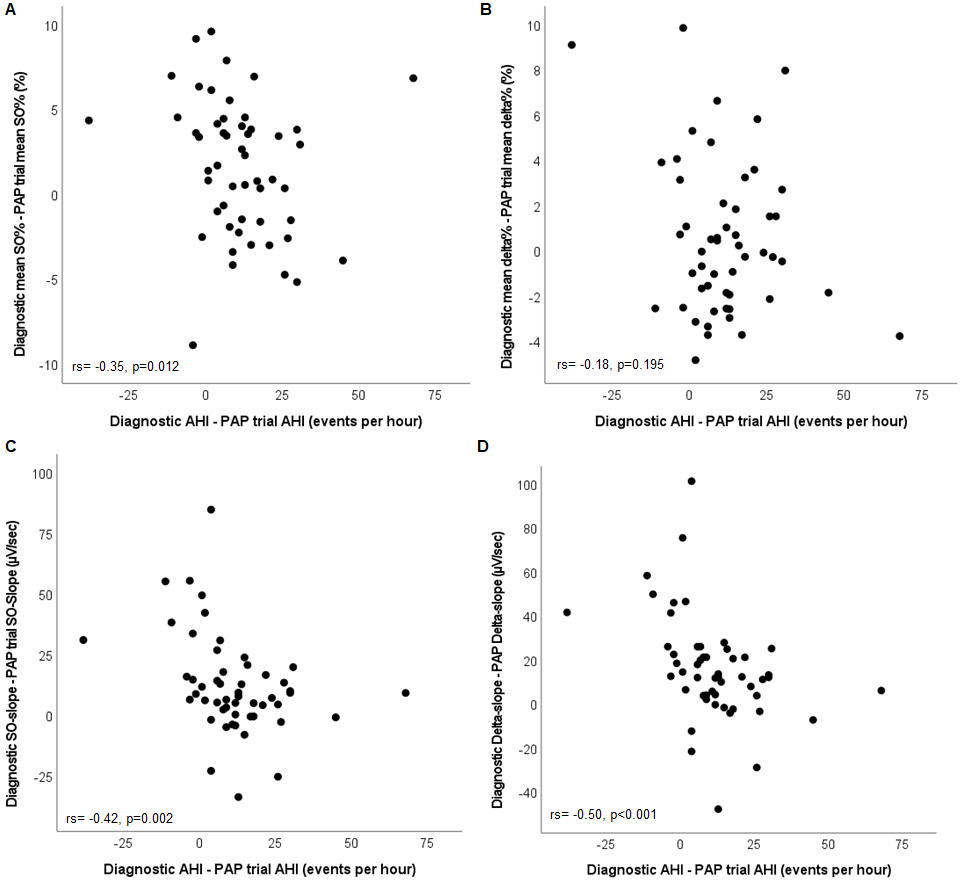


Supplementary Figure 1 legend. Unadjusted associations between difference in apnea-hypopnea indices (AHI) from diagnostic to positive airway pressure (PAP) trial portions of the sleep study and difference in mean non-rapid eye movement (NREM) slow oscillation (SO) power spectral density (%) **(A)**, difference in mean NREM delta power spectral density (%) **(B)** difference in mean NREM slow oscillation slopes (SO-slope) **(C)**, and difference in mean NREM delta slopes (delta-slope) **(D)** between diagnostic and PAP trial portions. Unadjusted correlation coefficients (rs: Spearman’s rank correlation coefficient) and statistical significance level is provided for each plot.

Supplementary Figure 2 heading: Associations between baseline NREM SWA features during early (diagnostic portion) and NREM SWA feature difference between diagnostic and PAP trial portions of the sleep study.


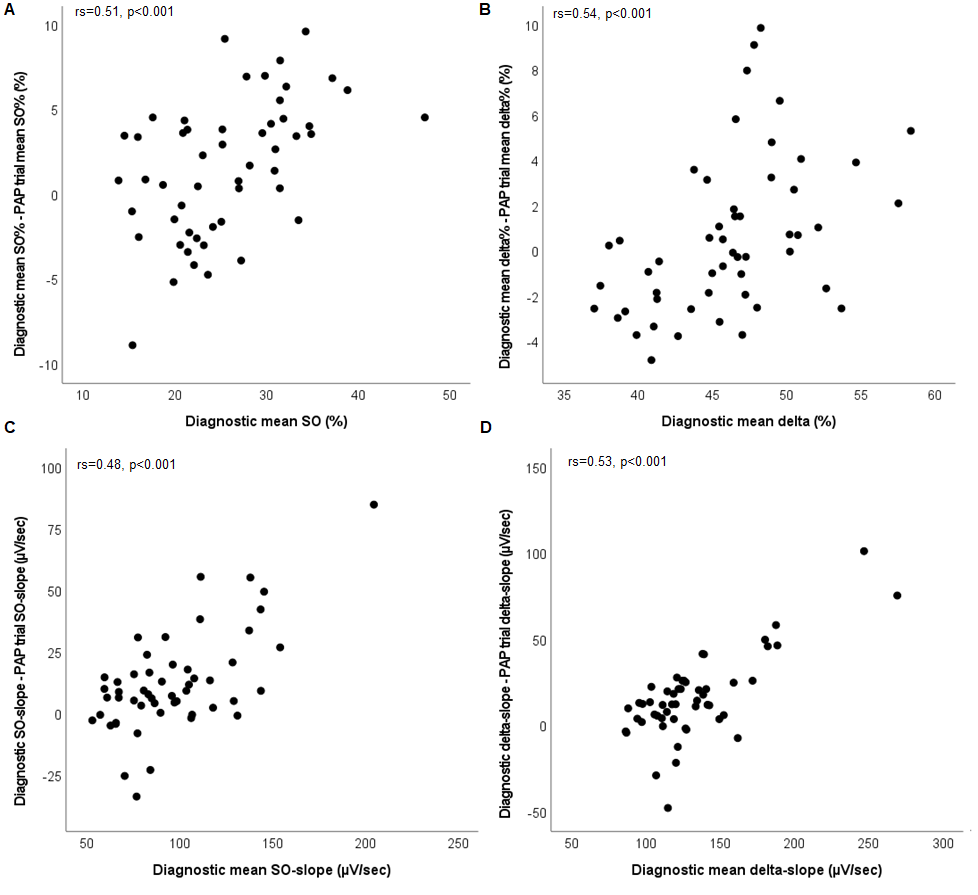


Supplementary Figure 2 legend. Unadjusted associations between baseline non-rapid eye movement (NREM) slow wave activity (SWA) features during diagnostic portion of the sleep study and its difference between diagnostic and positive airway pressure (PAP) portions for mean NREM slow oscillation power spectral density (%) **(A),** mean NREM delta power spectral density (%) **(B),** mean NREM slow oscillation slope (SO-slope) **(C)**, and mean NREM delta slope (delta-slope) **(D)**. Unadjusted correlation coefficients (rs: Spearman’s rank correlation coefficient) and statistical significance level (*P*) is provided for each plot.
